# Acute generalized exanthematous pustulosis induced by icotinib: a case report and literature review

**DOI:** 10.3389/fphar.2024.1462430

**Published:** 2024-08-30

**Authors:** Wei Yang, Jiayu Zhao, Jun Niu

**Affiliations:** Department of Dermatology, General Hospital of Northern Theater Command, Shenyang, China

**Keywords:** non-small cell lung cancer, tyrosine kinase inhibitor, icotinib, adverse drug reactions, acute generalized exanthematous pustulosis

## Abstract

Acute generalized exanthematous pustulosis, an infrequent adverse drug reaction, mainly results from drugs. Clinically, acute generalized exanthematous pustulosis manifests as a high fever, with skin lesions of small monomorphic subcorneal sterile pustules on an erythematous that presents at 1–4 days after medication exposure. The incidence of acute generalized exanthematous pustulosis varies from 3/1, 000, 000 to 5/1, 000, 000, while the mortality rate is typically around 5%. We present a case of a 69-year-old female who developed a diffuse, erythematous, pustular rash over the entire body and exhibited a fever of 38.3°C after 4 days of icotinib therapy. Considering her medication history and the appearance of the lesions, she was diagnosed with acute generalized exanthematous pustulosis and received appropriate treatment. We also conducted a literature review through PubMed to compare similarities and differences between our case and those reported in the literature.

## 1 Introduction

Icotinib is one of the epidermal growth factor receptor tyrosine kinases inhibitors (EGFR TKI), that reversibly inhibits EGFR signaling. Since it was initially made available in China, nearly 260,000 patients with non-small cell lung cancer (NSCLC) have been treated with icotinib. The most common adverse drug reactions of icotinib include rashes, dry skin, and diarrhea (He et al., 2021). However, reports of severe adverse drug reactions related to icotinib are relatively rare. Here, we report the first case of acute generalized exanthematous pustulosis (AGEP) induced by icotinib.

## 2 Case presentation

**FIGURE 1:**
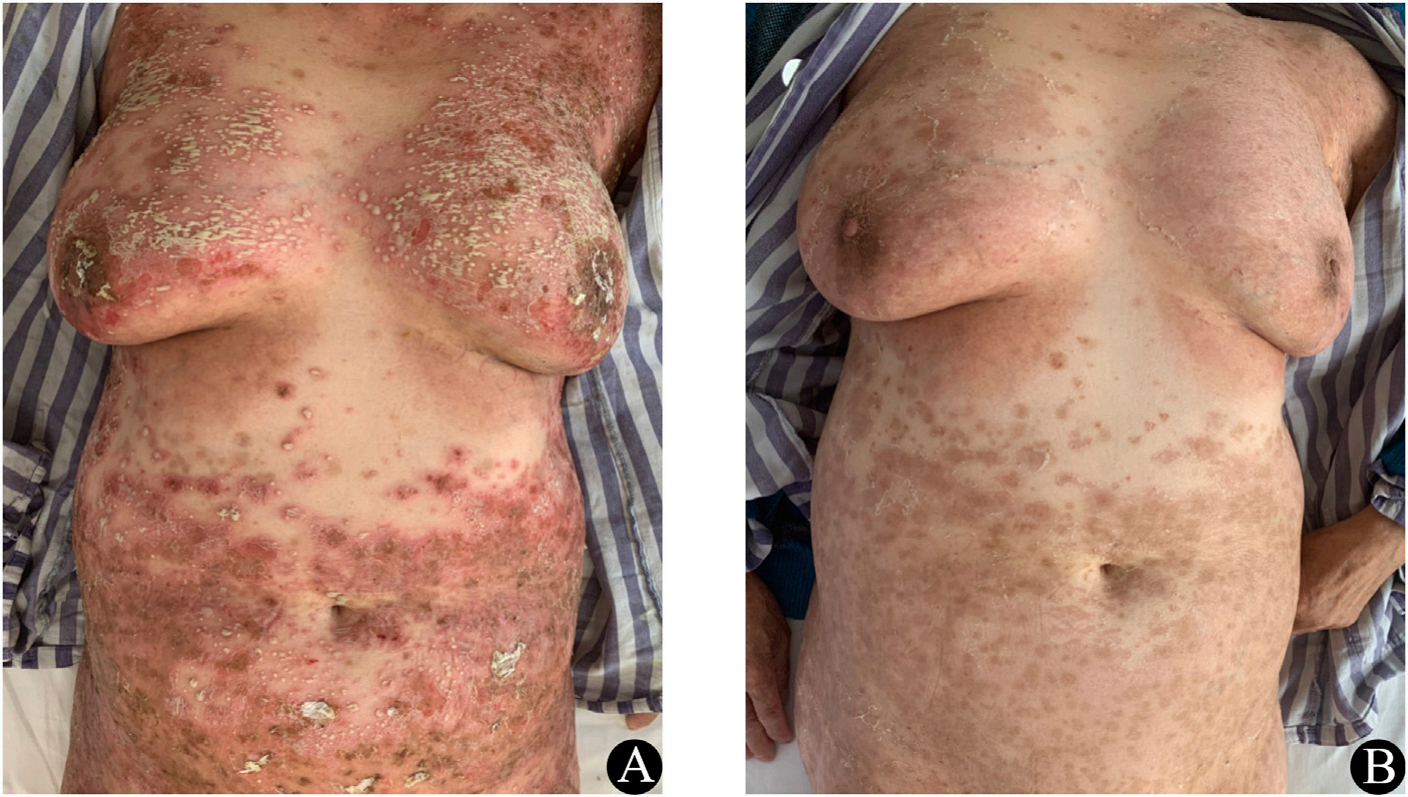
**(A)** Prior to treatment erythematous and pustular rashes were present on the trunk. **(B)** At 1 week after corticosteroid treatment, significant improvements in these cutaneous lesions were observed.

A 69-year-old Chinese female was diagnosed with left lung adenocarcinoma and diabetes. For her diabetes, she was taking oral sustained-release metformin tablets (1.5 g once daily) and acarbose tablets (50 mg thrice daily). For the lung adenocarcinoma, she received treatment with icotinib. She was not known to have any drug or food allergies. 4 days after receiving icotinib, she experienced a diffuse, erythematous, pustular rash over her entire body without mucosal involvement (Figure 1A) and a temperature of 38.9°C. Laboratory assay results revealed normal white blood cell counts (8.4 × 10^3^/µL with 72.8% neutrophils and 0.03% eosinophils), elevated acute-phase reactants (CRP 14.4 mg/dL, fibrinogen 5.19 g/L), and normal hepatic and renal functions. In light of her medication history and manifestation of lesions, AGEP was diagnosed. Icotinib was immediately discontinued and 40 mg of methyl-prednisolone was administered intravenously, with this dose subsequently reduced to 28 mg/d after 5 days. Following this methyl-prednisolone treatment, there was a lightening of the erythema, resolution of the pustules and widespread superficial desquamation was observed (Figure 1B). Within 2 weeks, the skin lesions had completely disappeared and the methyl-prednisone was gradually discontinued. Thereafter, the patient continued to take icotinib, experiencing only a few pustules, and was treated with halometasone/triclosan cream. 6 months later, due to the progression of the lung cancer, the patient was switched to osimertinib as the antineoplastic drug. 1 year later, the tumor had metastasized to the liver and bones. The patient underwent chemotherapy with pemetrexed and carboplatin, and was subsequently lost to follow-up 1 month after the treatment.

## 3 Discussion

AGEP is a severe cutaneous adverse reaction, which is most commonly triggered by exposure to certain drugs, including antibiotics, antifungals, and hydroxychloroquine (Szatkowski and Schwartz, 2015). Other risk factors encompass genetic factors, infections, and vaccinations (Szatkowski and Schwartz, 2015; Parisi et al., 2023). The diagnosis of AGEP is clinical, confirmed by histopathological examination (Hadavand et al., 2022). Differential diagnoses include autoimmune diseases such as generalized pustular psoriasis (GPP), IgA pemphigus, infectious diseases such as candida infection, and staphylococcal scalded skin syndrome (SSSS), as well as subcorneal pustular dermatosis (SPD). GPP is a form of psoriasis, with the gradual development of widespread and densely distributed sterile pustules on an erythematous base, accompanied by tenderness (Shi et al., 2022). IgA pemphigus is a specific type of pemphigus, characterized by blisters and pustules as the primary skin lesions in an autoimmune intraepidermal blistering disease. SSSS is an infection disease mediated by exfoliative toxins produced by coagulase-positive *Staphylococcus aureus*, and it is seen primarily in children. Candida infection usually manifests as erythematous, patches that are often accompanied by satellite papules and pustules. Intertriginous zones and the scrotum are most often involved and overall skin involvement is rare (Shepherd et al., 2017). SPD is an infrequent, benign, and recurrent pustular dermatosis, with remissions of variable duration. The pustules exhibit a superficial nature and are organized in annular and serpiginous configurations, particularly observed on the abdomen, axillae, and groins (Mohd Affandi and Baharom, 2023). For this reason, a cultured cutaneous secretion was included to exclude infectious diseases. Skin biopsy and direct immunofluorescence were not performed due to a lack of consent. The patient denied a family history or personal history of psoriasis, and based on her detailed medical history and typical manifestation of the lesion, we considered AGEP to be the most likely diagnosis. According to the AGEP validation score from the EuroSCAR study group, she was classified as a definite case, with an EuroSCAR score of 8 (Parisi et al., 2023).

Review of the literature revealed five cases of AGEP in patients with a reported EGFR-TKI exposure event from 2006 to 2024 (Shih et al., 2006; Lakshmi et al., 2010; Liquete et al., 2012; Komiya et al., 2021) (Table 1). We found that, four of six cases occurred in women. The time elapsed between medication exposure and the onset of the rash ranged from 4 days to 2 months. Five of these patients received corticosteroid treatment, and the rash resolved quickly. Interestingly, among the six patients, three were re-challenged with the culprit drug, resulting in limited pustular lesions. Recurrence of AGEP was observed in two patients subsequent to the resumption of the culprit drug. It is generally accepted that in the case of AGEP, a second episode may be more severe if the causative medication is readministered, therefore, avoiding further exposure is advised. However, for cancer patients experiencing cutaneous adverse reactions related to antineoplastic drugs, we need to collaborate with oncologists to grade the rash according to the Common Terminology Criteria for Adverse Events (CTCAE, Version 5.0). This assessment will help doctors decide if the patient can continue to take the causative drugs. The rash of this patient was classified as a grade 3 adverse event. It did not pose a threat to her life and resolved completely following appropriate treatment. Considering the diagnosis of stage IV lung cancer and the need for ongoing treatment, the decision was made to resume the medication with her informed consent. Although this patient exhibited only a few pustules after resuming the culprit drug and was able to control the symptoms with topical corticosteroids, it remains essential to monitor for any skin lesions in patients.

**TABLE 1:** Case report of AGEP associated with epidermal growth factor receptor tyrosine kinase inhibitors.

References	Country	Culprit drug	Sex	Age	Latency period	Therapy	Outcome
Shih et al. (2006)	United Kingdom	Gefitinib	Female	66	1 week	Prednisolone (30 mg/day)	Recovered
Shih et al. (2006)	United Kingdom	Gefitinib	Male	71	10 days	Prednisolone (30 mg/day)	Recovered
Lakshmi et al. (2010)	India	Lapatinib	Female	52	2 months	Topical corticosteroids antihistamine (amitryptiline)	Recovered
Liquete et al. (2012)	United States	Erlotinib	Male	63	<1 week	Steroids (unknown) Empiric antibiotics	Recovered
Komiya et al. (2021)	Japan	Erlotinib	Female	69	7 weeks	Prednisolone (1.0 mg/kg/day)	Recovered
Our case	China	Icotinib	Female	69	4 days	Methylprednisolone (40 mg/day)	Recovered

It has been well established that activated drug-specific T cells play a significant role in AGEP via a cascade of events (Mashiah and Brenner, 2003). Drugs form complexes with tissues in the body, stimulating the formation of drug-specific T cells and promoting their migration to the skin (Feldmeyer et al., 2016). Subsequently, CD8^+^ cells lead to the formation of subcorneal vesicles by releasing cytotoxic proteins and the CD4^+^ cells in these vesicles release IFN-γ, CXCL8 and GM-CSF. IFN-γ then promotes a further secretion of CXCL8 from surrounding keratinocytes, which effectively recruits neutrophils, while GM-CSF prevents apoptosis in these recruited neutrophils (Hadavand et al., 2022). Th17 cells, IL-17 and mutations in IL36RN may also be connected with AGEP (Hadavand et al., 2022). Mutations in the IL-36RN gene can lead to uncontrolled IL-36 signaling, which in turn stimulates the overproduction of additional proinflammatory cytokines and chemokines. This overproduction can exacerbate the recruitment and activation of neutrophils (Gabay and Towne, 2015). However, the pathogenesis of AGEP induced by EGFR-TKI remain unclear. EGFR is widely distributed in the skin, and skin-related adverse reactions are among the most common effects of EGFR-TKI therapy. Blockade of EGFR signaling downregulates CXCL8, while it increases CCL2, CCL5, and CXCL10 expression in keratinocytes, even under stimulation of IFN-γ(Mascia et al., 2003). In addition, the longest latency period observed in previous patients was nearly 2 months, and four of them continued the culprit drug treatment with only a few pustules observed, which differs from typical AGEP. Accordingly, we hypothesize that a different pathogenesis may underlie EGFR-TKI-induced AGEP, however, this hypothesis requires confirmation through further study.

The condition of most patients is generally self-limiting and the suspected drug must be discontinued immediately (Hadavand et al., 2022). Topical corticosteroids are used as supportive treatment, while systemic corticosteroids are used during the acute pustular phase or in severe cases (Hadavand et al., 2022; Parisi et al., 2023). Corticosteroids can quickly limit the inflammatory reaction and reduce the length of hospital stay (Oh et al., 2021). Accordingly, we used methylprednisolone (40 mg/d), her body temperature normalized within 24 h, the cutaneous lesions significantly improved within 5 days. And the dose of methylprednisolone was tapered to 0 within 2 weeks without any resurgence of symptoms. Besides, IL-17A inhibitors and IL-36 receptor inhibitors have been reported as a rapid and effective treatment for AGEP (Wen et al., 2024; Xuan et al., 2024). However, due to the presence of malignant tumors in our patient, treatment with IL-17A inhibitors and IL-36 receptor inhibitors was not considered in this case.

To the best of our knowledge, there have been no previously reported cases of icotinib-induced AGEP, however, current evidence suggests that EGFR-TKI is a rare cause of AGEP (Shih et al., 2006; Lakshmi et al., 2010; Liquete et al., 2012; Komiya et al., 2021). Furthermore, it has been implicated as a culprit drug in other severe cutaneous adverse reactions, including drug-induced hypersensitivity syndrome, Stevens-Johnson syndrome, and toxic epidermal necrolysis (Li et al., 2022). While EGFR-TKI have been widely used as the first-line treatment in NSCLC patients, it is important that any adverse events be reported in order to better understand the risks associated with this drug.

Limitations associated with this study should be noted. For example, the pervasiveness of these findings is limited by the small number of cases. And there is a lack of skin biopsy, patch test and lymphocyte transformation test in our case, all of which would be useful in establishing a definitive diagnosis.

## 4 Conclusion

EGFR-TKI is a rare cause of AGEP, with an uncertain incubation period. Corticosteroid therapy leads to a quick and complete resolution of skin lesions. While some culprit drugs could resume with only a few pustules being observed. In addition, the observation of skin lesions in patients receiving EGFR-TKI should be closely monitored.

## Data Availability

The original contributions presented in the study are included in the article/supplementary material, further inquiries can be directed to the corresponding author.
